# Metric validation for detection of delayed and directed coupling

**DOI:** 10.1088/1741-2552/ae5fd7

**Published:** 2026-05-14

**Authors:** Kate Dembny, Hafsa Farooqi, Alexander B Herman, Theoden I Netoff, David P Darrow

**Affiliations:** 1 Department of Biomedical Engineering, University of Minnesota, Minneapolis, MN, United States of America; 2 University of Minnesota, Medical Scientist Training Program, Minneapolis, MN, United States of America; 3 Department of Psychiatry, University of Minnesota, Minneapolis, MN, United States of America; 4 University of Minnesota, Medical Discovery Team on Addiction, Minneapolis, MN, United States of America; 5 Department of Neurosurgery, University of Minnesota, Minneapolis, MN, United States of America

**Keywords:** network neuroscience, effective connectivity, transfer entropy, delayed coupling, Granger causality, mutual information, cross-correlation

## Abstract

*Objective.* The brain functions as a complex network of billions of interconnected neurons, coordinating processes from basic reflexes to high-level cognition. Dysfunction in these networks contribute to neurological and psychiatric disorders, including epilepsy, depression, and Parkinson’s disease. Understanding these network alterations is essential for developing effective therapies. However, reconstructing network topology from human electrophysiology data is challenging due to sparse spatial sampling, measurement noise, and variable time delays in interregional communication. Effective connectivity (EC) metrics have been developed to infer directed neural interactions, but their accuracy under real-world data constraints remain unclear. This study empirically compares the ability of common EC metrics to reconstruct relationships between simulated time series with known temporal relationships and network topologies in the presence of data limitations common to human electrophysiology data. By utilizing networks and temporal relationships that are mathematically simple, this framework provides broad conceptual backing to understand the reliability of EC metrics and establishes groundwork upon which more complex spatial and temporal relationships between time series can be evaluated. *Approach.* We generated Erdős–Rényi networks and simulated time series using a time-delayed vector autoregressive model. We systematically varied network size, data length, measurement noise, and network coverage. Variations of four commonly used EC metrics, cross-correlation, Granger causality (GC), mutual information (MI), and transfer entropy, were evaluated for reconstruction accuracy using cosine distance, as well as receiver operating characteristic (ROC) curves, to compare estimated and true coupling matrices. *Main Results.* Multivariate transfer entropy demonstrated the highest accuracy across various conditions but required significantly longer computation times. For small networks (<30 nodes), MI and GC rapidly and accurately reconstructed networks. For larger networks, partial cross-correlation performed well with good computational efficiency. Notably, zero-lag metrics perform no better than chance for time-lagged time series relationships in nearly all conditions. *Significance.* The choice of an EC metric should consider specific data constraints. While multivariate transfer entropy is the most reliable across conditions, its long runtime limits its practical application. For large networks, partial cross-correlation offers a faster and reasonably accurate alternative. GC and MI are effective for small networks. Critically, time-lagged metrics are essential for accurate network reconstructions, as failing to account for time delays leads to reconstructions no more accurate than random network models.

## 1. Introduction

Neuroscience increasingly recognizes that cognitive functions arise from interactions between distributed brain regions rather than isolated activity in single areas (Bullmore and Sporns 2009, Bressler and Menon 2010, Park and Friston 2013, van den Heuvel and Sporns 2013, Beaty *et al* 2016). This shift in understanding is supported by studies identifying network-level dysfunction in various neurological and psychiatric disorders, such as altered frontostriatal circuit connectivity in schizophrenia that correlates with positive symptom severity (Gonzalez-Burgos *et al* 2011, Fornito *et al* 2013) and increased connectivity between default mode and salience networks in individuals with depression (Mulders *et al* 2015). Similarly, conditions such as Parkinson’s disease (Wu *et al* 2011) and traumatic brain injury (Sharp *et al* 2014), historically conceptualized as localized dysfunctions, are now understood to involve widespread network disruptions. These findings underscore the importance of inter-regional communication in understanding a broad variety of neurological and psychiatric disorders (Palop *et al* 2006, Fornito *et al* 2015, Vinogradov and Herman 2015). Fundamental to understanding network connectivity is the availability of metrics that measure communication and robustly and reliably identify these network connections. In practical application, the ability to identify these connections is complicated by the size of these networks, limited and noisy data, sparse sampling of brain areas, and physiological time lags in communication.

Determining the interactions between brain regions can be approached through causal manipulations, but these methods are often impractical or unethical in human studies. Instead, we rely heavily on observational data and a wide range of analytical methods to infer communication between regions, including anatomical, functional, and effective connectivity (EC). Anatomical connectivity maps the structural pathways along which communication may occur but does not capture the dynamic nature of brain activity (Lazar 2010). As a static model, anatomical connectivity cannot estimate dynamical changes in communication that occur over time, which are central to complex brain function (Sporns *et al* 2005). Functional connectivity, long a focus of functional MRI, can measure these dynamical relationships using statistical measures (Friston 2011) but fails to identify the directional influence necessary to understand causal relationships in complex brain networks. EC aims to infer directed interactions while accounting for physiological communication lags (Friston 2011, Lang *et al* 2012). These communication lags provide information about the directionality of connection based on the premise that causes precede their effects in time (Valdes-Sosa *et al* 2011). Consequently, EC metrics provide a powerful framework for understanding network dynamics, yet their accuracy depends on the methods used for estimation.

Numerous EC metrics have been proposed and individual metrics are well described throughout the literature (Zhou *et al* 2009, Vicente *et al* 2011, Wilmer *et al* 2012, Seth *et al* 2015, Shojaie and Fox 2022). However, each comes with inherent limitations that complicate interpretation. Despite the vast literature describing these metrics, and their widespread use in studying neural networks, no consensus has emerged on which metric most accurately reconstructs neural networks across varying conditions. A key challenge is that many studies apply EC metrics without validating their performance against known ground-truth networks. Additionally, real-world electrophysiology data often contain factors that can distort connectivity estimates, including sparse sampling, measurement noise, and limited data length. Some existing literature investigates the accuracy of these metrics, but several limitations predominate. Most compare the performance of metrics in only a single condition (Kim *et al* 2013), only look at the performance of a single metric (Ursino *et al* 2020), compare small methodological differences between two versions of the same metric (Barnett and Seth 2014), or fail to examine the time lagged relationship between signal processes (David *et al* 2004). Bastos and Schoffelen have one of the most cited papers looking at several methods, and while they outline potential pitfalls of specific metrics, they do not empirically compare metrics to one another (Bastos and Schoffelen 2016). We seek to address these gaps, by systematically evaluating commonly used EC metrics, and testing their ability to reconstruct simulated networks under conditions that mimic typical electrophysiological constraints. These experiments allow us to identify how well each metric performs and identify optimal conditions for use. By comparing how well each metric performs across different conditions, we aim to identify optimal methods for reconstructing brain networks and improve the reliability of connectivity-based neuroscience research.

## 2. Methods

Consider the brain network with regions identified as nodes ${{\tilde N}} = \{ 1, \ldots ,{{N}}$ }, and the behavior of each node, represented by a Gaussian stationary time series, $X = \left\{ {{x_1}, \ldots ,{x_{\mathrm{N}}}} \right\}$. For the electrophysiological signal associated with each region, we discretize time with the sampling time ${T_{\mathrm{s}}}$ into discrete time samples $k$. Therefore, the actual time $t$ at each sampling instant is equal to $t = k{T_{\mathrm{s}}}$. Since signals are defined for finite time, we represent the final time sample with ${k_{\mathrm{f}}}$, such that $k = 0, \ldots ,{k_{\mathrm{f}}} - 1$.

### 2.1. Network simulations

In this study, we simulate networks with time-lagged communication between nodes. Each simulated network is represented by a connectivity tensor ${A_{{\mathrm{m}},{\mathrm{n}},{\mathrm{l}}}} \in {\mathbb{R}^{N \times N \times L}}$, where $m$ and $n$ are any two nodes communicating with each other and ${\mathrm{l}}$ is the lag of this communication within a maximum number of possible lags, $L$. A random number generator is used to create a connectivity tensor with a 10% coupling probability between any two nodes, resulting in sparse and generally stable networks with Erdős–Rényi topologies. Erdős–Rényi models are characterized by two assumptions—that each potential connection is equally likely, and that each of these connections is independent. Though this topology is not representative of typical brain network structures, an Erdős–Rényi topology was selected for a number of reasons. Properties of Erdős–Rényi network topologies are conceptually simple and well characterized mathematically. Additionally, while topology may be an important consideration for understanding these networks, an infinite number of topological permutations are possible, and as a result cannot be exhaustively searched. Therefore, we selected a simplistic and well-understood topological model as a baseline. Future iterations on this work could include investigation of topological variants seen in the literature, such as scale-free small-world networks (Watts and Strogatz 1998), or second-order network relationships (Zhao *et al* 2011).

Given this topology, we simulated time series data using damped coupled oscillators with time delays representing the interregional communication. Each node’s activity was modeled as a first-order coupled oscillator, incorporating lags to reflect realistic transmission delays between brain regions. This first-order relationship was selected to model a clearly identifiable linear relationship between time series and is common to other models in the literature (Bick *et al* 2020, Nozari *et al* 2024). While relationships between neurophysiological time series are plausibly more complex, this model simulates a simple relationship that metrics should be able to identify. If they cannot, they are unlikely to successfully identify more complex relationships. This system is governed by the following equation for a damped harmonic oscillator (Dekker 1981):

\begin{align*}{x_m}\left[ k \right] &amp; = 2{\mathrm{DS}}{x_m}\left[ {k - 1} \right] - {D^2}{x_m}\left[ {k - 2} \right] \nonumber\\ &amp; \quad + {{\zeta }}\left[ k \right] - {{DS\zeta }}\left[ {k - 1} \right] + \mathop \sum \limits_{j \in \tilde N} \mathop \sum \limits_{l = 1}^{{L_{m,j}}} {A_{m,j,l}}{x_j}\left[ {k - l} \right].\end{align*}

**Figure 1.**
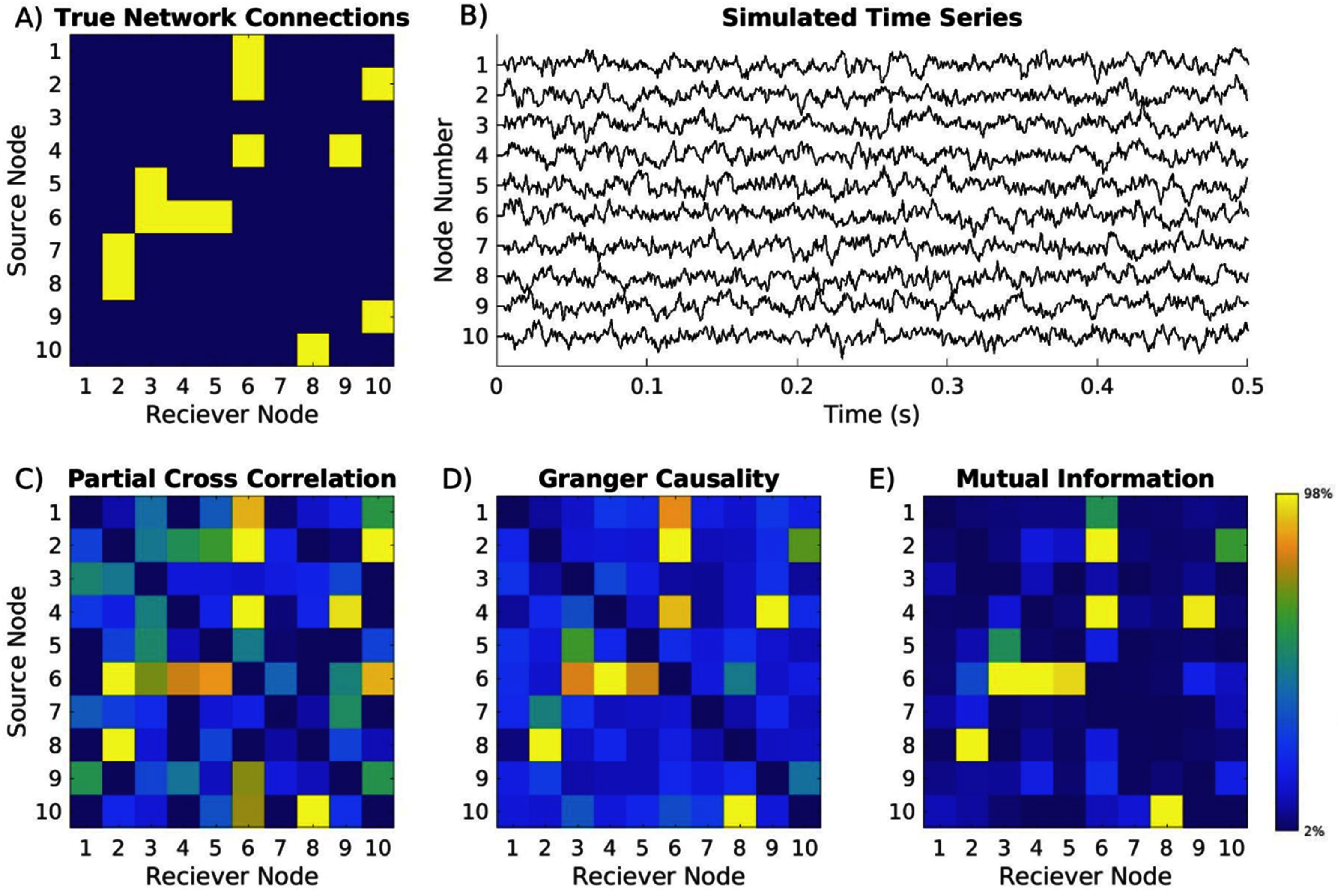
Example of oscillatory networks and reconstructions with common effective connectivity metrics. (A) Adjacency matrix showing randomly generated network connectivity model, where yellow indicates nodes are connected; (B) simulated time series where nodes generate theta frequency oscillations, and connectivity is determined by selected adjacency matrix in (A) (amplitude in arbitrary units); (C) calculated effective connectivity of time series from (B) using partial cross-correlation; (D) Granger causality; and (E) mutual information. Color bar is scaled from 2nd to 98th percentile of all network values for each metric. In this single example, effective connectivity metrics show different abilities to reconstruct network relationships, with partial cross correlation producing a reliable but noisy reconstruction, and mutual information producing a similar general structure, but one that is less noisy than the partial cross correlation values.

Where $D = {{\mathrm{e}}^{ - {{\mathrm{T}}_{\mathrm{s}}}/{{\tau }}}}$ a decay term with time constant $\tau $, and $S = \cos \left( {{\omega _0}k{T_{\mathrm{s}}}} \right)$ determines the natural frequency of oscillation with the center frequency at. ${{\zeta }}\left( t \right)$ is a Gaussian distributed random number generator, and ${L_{m,j}}$ is the lag between node ${\mathrm{m}}$ and any node ${\mathrm{j}} \in \tilde N$, which is a randomly selected integer between 1 and 10 timesteps. An exemplar oscillatory system is shown in figure 1 with the known coupling matrix, a binarization of $A$ that notates whether two nodes are communicating across any lag, simulated time series, and several EC reconstructions using several metrics described below.

While coupled oscillator models provide a biologically plausible framework for simulating network dynamics, they require predefined center frequencies and time delays, which can introduce additional complexity. Additionally, the presence of autocorrelations in these systems increases the variance in cross-correlation estimates, necessitating longer time series for statistically robust results (Jenkins and Watts 1969). To circumvent these challenges, we implemented a vector autoregressive (VAR) model without autocorrelations. While VAR models are simplistic, they generate clearly identifiable linear relationships between time series. These simplistic relationships should be detectable by most metrics. If metrics cannot successfully reconstruct these networks they are unlikely to reconstruct networks with more complex relationships. In this model, each node’s activity is driven by independent and identically distributed (i.i.d.) noise, with short communication lags ranging from 1 to 10 time steps. The coupling strength between nodes was held constant to ensure consistency across simulations. An i.i.d. time series was selected because preprocessing of neural time series data traditionally begins with whitening to remove auto-correlations. The presence of strong autocorrelations in two independent processes can induce the appearance of EC between them, even when these signals are not communicating (Dean and Dunsmuir 2016). As a result, we designed our sample time series to be i.i.d. to avoid the presence of autocorrelations in the data and reflect this standard preprocessing step common in neural data analysis.

To establish this model, we assume that any time series signal ${x_m} \in X$ has an equivalent $L{\mathrm{th}}$ order VAR model of the form:

\begin{equation*}\begin{array}{*{20}{c}} {{x_m}\left[ k \right] = \mathop \sum \limits_{j \in \tilde N} \mathop \sum \limits_{l = 1}^{{L_{m,j}}} {A_{m,j,l}}{x_j}\left[ {k - l} \right] + {\zeta _d}\left[ k \right]} \end{array}\end{equation*}

where $A$ is a three-dimensional tensor of coefficients representing the coupling between node $m{\text{ }}$ and $j$ at each possible lag $l$, where ${L_{m,j}}$ is the maximum lag between the nodes $m$ and any node $j$, and ${{{\zeta }}_{\mathrm{d}}}$ is the dynamical noise determined by a normally distributed source with standard deviation ${\sigma _{\mathrm{d}}}$. The self-coupling is zero at all lags ${A_{m,m,l}} = 0,\,\,{\mathrm{l}} = 1, \ldots ,{L_{{\mathrm{mm}}}}$. For the coupling drive weights from node $m$ onto node $n$, we use value 0 for no communication between the nodes, and $\{ 1, - 2\} $ for possible communication between any two nodes. These values are sequentially introduced into the connectivity tensor as $\left[ {1, - 2,1} \right]$, which comes from the second temporal derivative of the activity of node $j$, to simulate a coupling relationship that exceeds the complexity of a simple binary connected/not-connected model. This approach ensures stable network dynamics while maintaining a realistic representation of time-lagged interactions.

To simulate measurement noise, we introduced an independent noise component to each node’s signal:

\begin{equation*}{\overset{\lower0.5em\hbox{$\smash{\scriptscriptstyle\frown}$}}{x} _m}\left[ k \right] = {x_m}\left[ k \right] + {{{\zeta }}_{{\mu }}}\left[ k \right]\end{equation*}

where ${x_m}$ is the state of the system at node $m$, and ${\overset{\lower0.5em\hbox{$\smash{\scriptscriptstyle\frown}$}}{x} _m}$ is the measured signal, including measurement noise ${{{\zeta }}_\mu }$, a normal distribution with mean zero and standard deviation ${\sigma _\mu }$. Simulations were generated in mathworks 2023a. All code used for the project can be found at https://github.com/hermandarrowlab.

**Figure 2.**
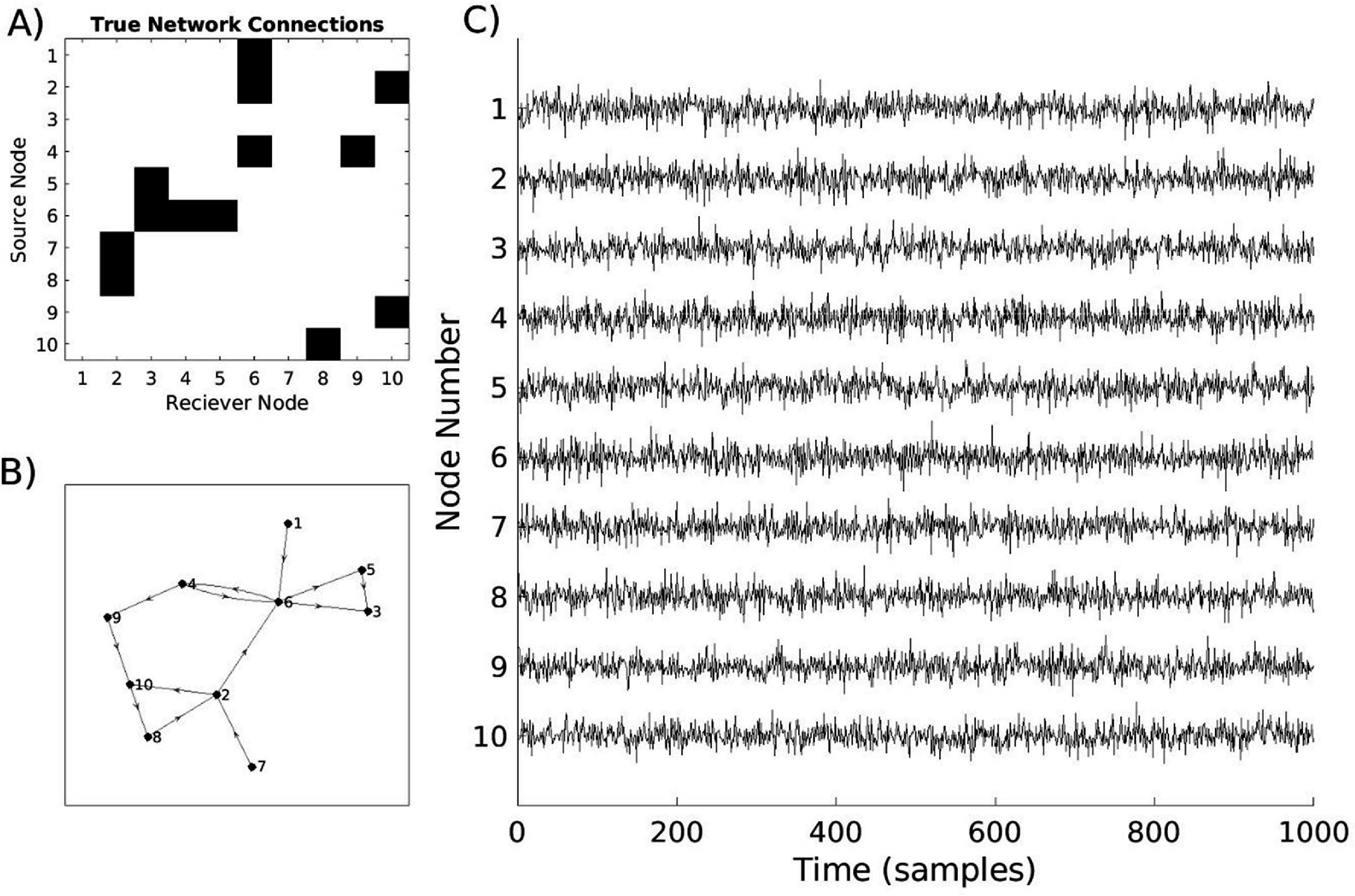
Sample of network and time series data generated by model—(A) randomly generated coupling matrix of network connectivity; (B) visual representation of topology of the network in (A), with directional arrows representing directed connection; (C) sample time series for 10-node network with time-lagged connections generated by VAR model using coupling matrix in (A) (amplitude in arbitrary units). This example demonstrates the general procedure used to create the simulated datasets for the following analyses.

**Figure 3.**
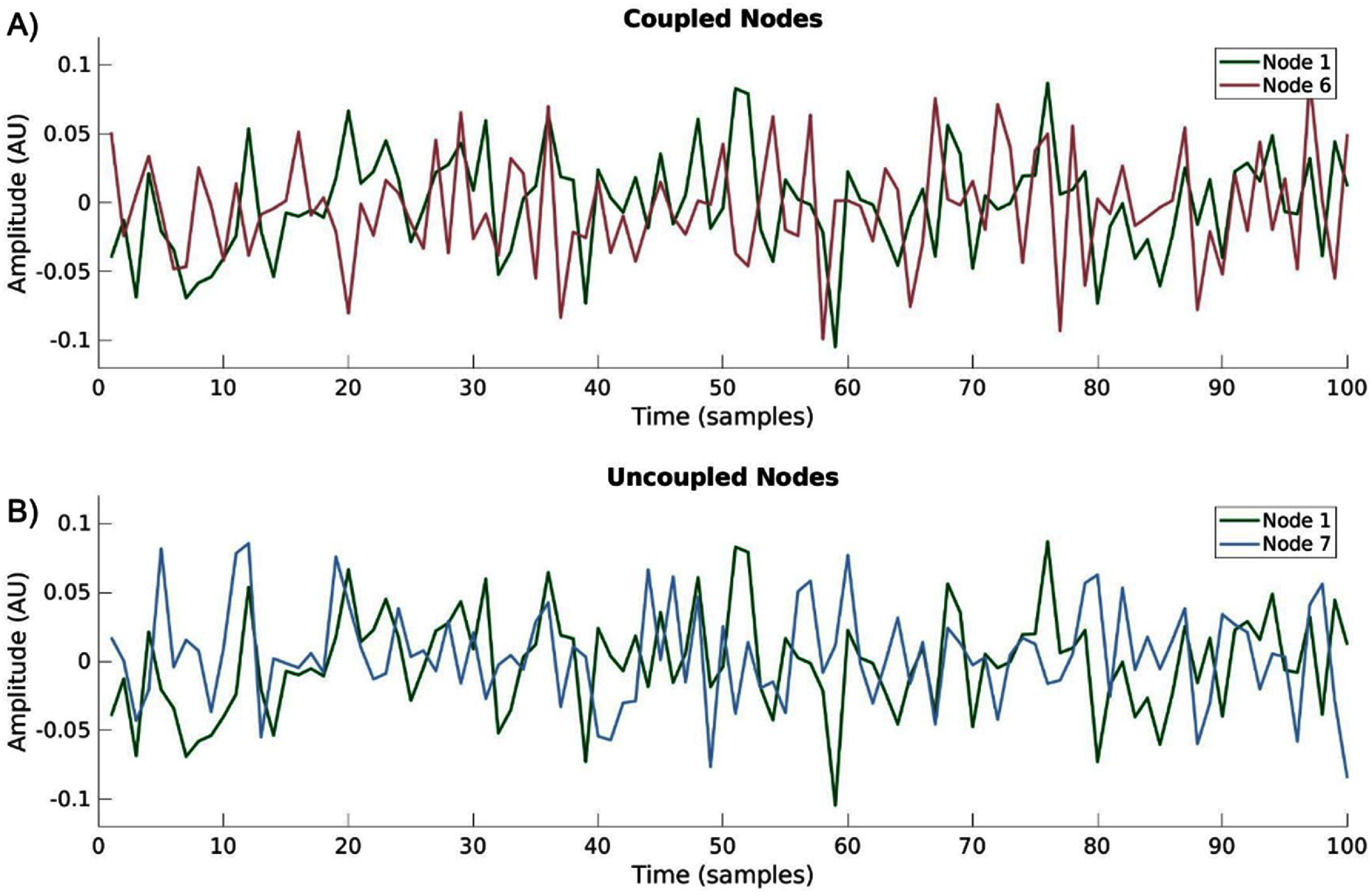
Channel coupling is not visually identifiable in coupled versus uncoupled nodes—(A) example of time series of two coupled nodes from network seen in figure 2 with amplitude in arbitrary units (AU); (B) example of time series of two uncoupled nodes from network seen in figure 2 with amplitude in arbitrary units. These sample time series demonstrate the coupled nodes are not readily visually identifiable as different from non-communicating time series.

To illustrate the simulated networks, we generated an example 10-node network and visualized its connectivity structure. The coupling matrix (figure 2(A)), is a binarized form of $A$, the coupling tensor, which represents whether directed interactions between nodes occur at any lag. In this array, nonzero entries indicate the presence of directed coupling between the two nodes. A topological representation of this network is shown in figure 2(B), where arrows denote the direction of information flow between nodes. Time series data were generated for each node using the VAR model in equation (1), incorporating predefined coupling delays and measurement noise (figure 2(C)). In these simulations, coupling strength was set at a level where inter-node communication was not visually apparent in the raw time series, ensuring that connectivity could not be inferred through simple visual inspection (figure 3). Instead, EC metrics were required to reconstruct the underlying network structure, allowing for an objective evaluation of their performance.

### 2.2. Parameter variation

Four model parameters were varied when generating time series data (table 1). The variations of these parameters model conditions that limit network reconstruction reliability in intracranial electrophysiology data. These parameters include:

**Table 1.** Parameter variation this table identifies parameter sets used to generate each set of simulated data when experimental parameters are varied.

	Conditions for parameter varied				
		Nodes	Time points	SNR	Network coverage
Experimental Parameter	Number of nodes	10, 20, 30, 50, 65	10 000	No noise	100%
	Number of time points	50	500, 1000, 5000, 10 000	No noise	100%
	SNR	50	10 000	0.01, 0.05, 0.1, 0.5, 1, 5, 10, 50	100%
	Network coverage	50	10 000	No noise	100%, 90%, 80%, 70%, 60%, 50%, 40%, 30%, 20%, 10%

*Number of nodes (*$N$): Networks with 10, 20, 30, 50, and 65 nodes were generated. When other parameters were varied, the number of nodes used for the simulation was held constant at 50.

*Number of data points (*$k$): The number of data points in the time series was varied with options of 500, 1000, 5000, and 10 000. The number of time points was held constant at 10 000 when other parameters were varied.

*Measurement noise (*$U$): Independent white measurement noise was added to each node as a function of signal-to-noise ratio (SNR) using Matlab’s awgn function. SNR values were 0.01, 0.05, 0.1, 0.5, 1, 5, 10, and 50. Simulations contained no noise when other parameters were varied.

*Percent network coverage:* Networks were simulated with all of the default parameters above. A pseudorandom number generator selected 5 nodes (10% of the network) and their time series to exclude from the network and EC calculations. This was done iteratively until only 5 nodes remained in the network (10% network coverage). Network coverage was held at 100% when other parameters were varied.

### 2.3. Network reconstruction metrics

In this work, we evaluate the ability of standard EC metrics to identify time-lagged and directed communication from time series data. We focus on four metrics: cross-correlation, Granger causality, (GC) mutual information (MI), and transfer entropy. All of these methods have implementations available as packages in Matlab or Python. For each lagged metric, maximum lag calculated was set at the 10 lags, concordant with the maximum lags simulated in the model. Below, we briefly define the mathematical formulations for each metric.

Consider two measured time series brain signals ${\overset{\lower0.5em\hbox{$\smash{\scriptscriptstyle\frown}$}}{x} _m}$ and ${\overset{\lower0.5em\hbox{$\smash{\scriptscriptstyle\frown}$}}{x} _n}$, where ${\mu _{\widehat{{{{x}}_{\mathrm{m}}}}}}$ and ${\mu _{\widehat{{{{x}}_n}}}}$ are the mean of the signals ${\overset{\lower0.5em\hbox{$\smash{\scriptscriptstyle\frown}$}}{x} _m}$ and ${\overset{\lower0.5em\hbox{$\smash{\scriptscriptstyle\frown}$}}{x} _n}$, respectively, and ${{\sigma }}_{\widehat{{x_m}}}^2$ and ${{\sigma }}_{\widehat{{x_n}}}^2$ are their variances. When signals ${\overset{\lower0.5em\hbox{$\smash{\scriptscriptstyle\frown}$}}{x} _m}$ and $\widehat{{{\mathrm{x}}_{\mathrm{n}}}}$ are excluded from the analysis, we denote the remainder network as $\widetilde {{{\mathrm{N}}_{\mathrm{r}}}} = {{\tilde N}} \setminus \left\{ {{\mathrm{m}},{\mathrm{n}}} \right\}$ and the remainder time series as $\widehat{{X_{\mathrm{r}}}} = \hat X \setminus \left\{ {{{\overset{\lower0.5em\hbox{$\smash{\scriptscriptstyle\frown}$}}{x} }_m},\widehat{{x_n}}} \right\}$.

Cross-correlation: Correlation is a well-established measure that estimates the linear relationship between two signals and is robust even in noisy conditions (Netoff *et al* 2004, Ostojic *et al* 2009, Rodu *et al* 2018). Cross-correlation can be measured between two signals at different time delays to identify communication lag. These lags provide information about communication direction given the assumption that causes precede their effects. However, there are limitations. Cross-correlation assumes linear relationships between time series, so it may miss nonlinear relationships (Sakkalis 2011) with no significant linear component. Additionally, the metric has difficulty identifying bidirectional interactions and struggles to interpret multiple delays with high correlation values (Bastos and Schoffelen 2016).

*Bivariate cross-correlation:* In the time domain, the cross-correlation of $\widehat{{x_m}}$ and $\widehat{{x_n}}$ at lag $l$ can be calculated using the following mathematical formulation:

\begin{equation*}{{C}}_{{{{c}}_{{{mn}}}}}^{{{\hat x}}}\left[ {{l}} \right] = \frac{{{{C}}_{{{{v}}_{{{mn}}}}}^{{{\hat x}}}\left[ {{l}} \right]}}{{\sqrt {{{\sigma }}_{\widehat{{{{x}}_{{m}}}}}^2{{\sigma }}_{\widehat{{{{x}}_{{n}}}}}^2} }}\end{equation*}

where covariance $C_{{v_{mn}}}^{\hat x}\left[ l \right]$ between the brain signals $\widehat{{x_m}}$ and $\widehat{{x_n}}$ is further defined as:

\begin{equation*}C_{{v_{mn}}}^{\hat x}\left[ l \right] = \frac{1}{{{k_f} - l}}\mathop \sum \limits_{k = l}^{{k_f} - 1} \left( {\widehat{{x_m}}\left[ {k - l} \right] - {{{\mu }}_{\widehat{{x_m}}}}} \right)\left( {\widehat{{x_n}}\left[ k \right] - {{{\mu }}_{\widehat{{x_n}}}}} \right).\end{equation*}

The cross-correlation is equivalent to normalized cross-covariance. For each possible pair of nodes, the maximum cross-correlation value across all possible lags was extracted and used as the estimate to be compared against the true coupling matrix.

*Partial cross-correlation:* Within a time series, it is important to identify whether similar recorded signals result from communication between two connected regions or, alternatively, if the two signals have a shared source with which they both independently communicate. In an attempt to resolve this conflict, partial cross-correlation has been proposed.

When calculating the partial lagged cross-correlation in the time domain, information contained in, can be used to estimate the best linear predictor $\widetilde {{x_m}}$ of the signal $\widehat{{x_m}}$ that minimizes the residual ${ \epsilon _m} = {\hat x_m} - {\tilde x_m}$ and $\widetilde {{{\mathrm{x}}_{\mathrm{n}}}}$ of the signal $\widehat{{x_n}}$ that minimizes the residual ${ \epsilon _n} = {\hat x_n} - {\tilde x_n}$. The partial lagged cross-correlation of the time series signals $\widehat{{x_m}}$ and $\widehat{{x_n}}$ is defined as the lagged cross-correlation between the residuals ${ \epsilon _m}$ and ${ \epsilon _n}$.

In practice, however, it is difficult to identify accurate estimators of $\widehat{{x_m}}$ and $\widehat{{x_n}}$ in the time domain. We instead calculate cross-correlation in the frequency domain, where the need to calculate these estimators is avoided. To accomplish this frequency domain calculation, for any frequency ${{\lambda }} \in \left[ { - {{\pi }},{{\pi }}} \right]$, we define the Discrete-Time Fourier transform of the covariance between the time series $\widehat{{x_m}}$ and $\widehat{{x_n}}$ as:

\begin{equation*}{f_{mn}}\left( \lambda \right) = \frac{1}{{2\pi }}\mathop \sum \limits_{l = - \infty }^\infty C_{{v_{mn}}}^{\hat x}\left[ l \right]{{\mathrm{e}}^{ - i{{\lambda }}l}}.\end{equation*}

Next, the partial cross-spectral density between the signals is defined as:

\begin{equation*}f_{mn}^{\,p}\left[ {{\lambda }} \right] = \frac{{ - {f_{mn}}{{\left( {{\lambda }} \right)}^{ - 1}}}}{{{f_{mm}}{{\left( {{\lambda }} \right)}^{ - 1}}{f_{nn}}{{\left( {{\lambda }} \right)}^{ - 1}} - {{\left| {{f_{mn}}{{\left( {{\lambda }} \right)}^{ - 1}}} \right|}^2}}}\end{equation*}

and the partial covariance $PC_{{v_{mn}}}^{\hat x}\left[ l \right]$ is calculated using the inverse discrete Fourier transform:

\begin{equation*}{{PC}}_{{{\mathrm{v}}_{{\mathrm{mn}}}}}^{{{\hat x}}}\left[ {\mathrm{l}} \right] = \mathop \int \limits_{ - {{\pi }}}^{{\pi }} {\mathrm{f}}_{{\mathrm{mn}}}^{\mathrm{p}}\left( {{\lambda }} \right){{\mathrm{e}}^{{{i\lambda l}}}}{\mathrm{d}\lambda }.\end{equation*}

In summary, ${{PC}}_{{{\mathrm{v}}_{{{mn}}}}}^{{{\hat x}}}\left[ {\mathrm{l}} \right]$ can be calculated from cross-spectral densities ${f_{mn}}\left( {{\lambda }} \right)$ of the simple lagged cross-correlations between $\widehat{{x_m}}$ and $\widehat{{x_n}}$ (Eichler *et al* 2003, Salvador *et al* 2005). As in bivariate cross-correlation, for each possible pair of nodes, the maximum cross-correlation value across all possible lags was extracted and used as the estimate to be compared against the true coupling matrix. Cross-correlation was calculated using the Functional Connectivity Toolbox (Zhou *et al* 2009).

GC: GC estimates the reduction in forecasting error resulting from including the history of an additional node in an autoregressive model used to estimate the next time point (Granger 1969, Lütkepohl 2005). Initially proposed for economics data, the metric is broadly utilized within neuroscience literature (Barnett and Seth 2014, Shojaie and Fox 2022). However, GC assumes the recorded time series offers a complete representation of the system, which is often untrue in neurophysiology recordings (Shojaie and Fox 2022). While the original GC metric assumes linear relationships, nonlinear and nonparametric extensions have been developed (Dhamala *et al* 2008, Barnett and Seth 2014, Sysoeva *et al* 2014, Zhang *et al* 2016).

Now consider signals $\widehat{{x_n}}$ and $\widehat{{x_m}}$. In order to calculate GC between two time series, we define the full $\widehat{x_m^f}\left[ k \right]$ and reduced $\widehat{x_m^r}\left[ k \right]$ regression models of the time-series signal $\widehat{{x_m}}$:

\begin{align*}\widehat{{\mathrm{x}}_{\mathrm{m}}^{\mathrm{f}}}\left[ {\mathrm{k}} \right] &amp; = \mathop \sum \limits_{l = 1}^{{L_{m,m}}} {A_{m,m,l}}\widehat{{x_m}}\left[ {k - l} \right] + \mathop \sum \limits_{l = 1}^{{L_{m,n}}} {A_{m,n,l}}\widehat{{x_n}}\left[ {k - l} \right] \nonumber\\ &amp; \quad + \mathop \sum \limits_{l = 1}^{{L_{m,{{\eta }}}}} \mathop \sum \limits_{{{\eta }} \in \widetilde {{N_r}}} {A_{m,{{\eta }},{\mathrm{l}}}}\widehat{{x_{{\eta }}}}\left[ {k - l} \right] + R_m^f\left[ k \right]\end{align*}

\begin{align*}\widehat{{\mathrm{x}}_m^{\mathrm{r}}}\left[ {\mathrm{k}} \right] &amp; = \mathop \sum \limits_{l = 1}^{{L_{m,m}}} {A_{m,m,l}}\widehat{{x_m}}\left[ {k - l} \right] \nonumber\\ &amp; \quad + \mathop \sum \limits_{l = 1}^{{L_{m,{{\eta }}}}} \mathop \sum \limits_{{{\eta }} \in \widetilde {{N_r}}} {A_{m,{{\eta }},{\mathrm{l}}}}\widehat{{x_{{\eta }}}}\left[ {k - l} \right] + R_m^r\left[ k \right]\end{align*}

where tensor $A$ contains the regression coefficients for the appropriate nodes, and $R_m^f\left[ k \right]$ and $R_m^r\left[ k \right]$ are the residuals for the full model and the reduced model respectively at time sample $k$. If the inclusion of $\widehat{{x_n}}$ reduces the residual, it can be concluded that the $\widehat{{x_n}}$ is sending directed communication to $\widehat{{x_m}}$. Having defined the appropriate models, we define multivariate GC from $\widehat{{x_n}}$ to $\widehat{{x_m}}$ conditioned on $\widehat{{x_r}}$ as the following log-likelihood ratio:

\begin{equation*}{\text{ }}{{\mathrm{G}}_{{{\mathrm{c}}_{{\mathrm{n}} \to {\mathrm{m}}|\widehat{{{\mathrm{x}}_{\mathrm{r}}}}}}}} \equiv \ln \frac{{\left| {C_{{v_{mn}}}^{{R^r}}\left[ l \right]} \right|}}{{\left| {C_{{v_{mn}}}^{{R^f}}\left[ l \right]} \right|}}\end{equation*}

where $C_{{v_{mn}}}^{{R^r}}\left[ l \right]$ and $C_{{v_{mn}}}^{{R^f}}\left[ l \right]$ are the cross-covariances of the residuals, as seen in the definition of cross-correlation. GC was calculated using the Multivariate Granger Causality Toolbox by Anil Seth (Barnett and Seth 2014).

MI: MI has a mathematical basis in Shannon entropy, which estimates information flow between two signals using their probability distributions (Grassberger *et al* 1991, Emmert-Streib and Dehmer 2009). As a symmetric operator, MI calculates coupling without directionality. However, time-lagged versions have been developed by calculating MI while shifting the temporal alignment between signal processes (Schreiber 2000), and one such implementation is used in this paper. In time series data, the assumption of independence between time samples is invalid since time series often show temporal correlations. To account for this, we assume each sampled pair ${x_m}\left[ k \right]$, ${x_n}\left[ k \right]$ is a random variable for every time sample. Under this assumption, we define MI as (Shannon 1948, Schreiber 2000):

\begin{equation*}{\mathrm{M}}{{\mathrm{I}}_{{\mathrm{mn}}}} = \mathop \sum \limits_{{\mathrm{k}} = 0}^{{{\mathrm{k}}_{\mathrm{f}}} - 1} {\mathrm{p}}\left( {\widehat{{{\mathrm{x}}_{\mathrm{m}}}}\left[ {\mathrm{k}} \right],\widehat{{{\mathrm{x}}_{\mathrm{n}}}}\left[ {\mathrm{k}} \right]} \right){\log _{10}}\frac{{p\left( {\widehat{{x_m}}\left[ k \right],\widehat{{x_n}}\left[ k \right]} \right)}}{{p\left( {\widehat{{x_m}}\left[ k \right]} \right)p\left( {\widehat{{x_n}}\left[ k \right]} \right)}}.\end{equation*}

The notation ${\mathrm{p}}\left( {\widehat{{x_m}}\left[ k \right],\widehat{{x_n}}\left[ k \right]} \right)$ denotes the joint probability density function of $\widehat{{x_m}}$ and $\widehat{{x_n}}$, while

$p\left( {\widehat{{x_m}}\left[ k \right]} \right)$ and $p\left( {\widehat{{x_n}}\left[ k \right]} \right)$ are the marginal probability density functions of $\widehat{{x_m}}$ and $\widehat{{x_n}}$ respectively at sample $k$. For lag $l$, we define the lagged MI as:

\begin{align*}{\mathrm{M}}{I_{mn}}\left[ l \right] &amp; = \mathop \sum \limits_{k = l}^{{k_f} - 1} p\left( {\widehat{{x_m}}\left[ {k - l} \right],\widehat{{x_n}}\left[ k \right]} \right){\log _{10}} \nonumber\\ &amp; \quad \times \frac{{p\left( {\widehat{{x_m}}\left[ {k - l} \right],\widehat{{x_n}}\left[ k \right]} \right)}}{{p\left( {\widehat{{x_m}}\left[ k \right]} \right)p\left( {\widehat{{x_n}}\left[ k \right]} \right)}}.\end{align*}

As with cross-correlation metrics, the maximum MI across all possible lags was extracted and used as the estimate to be compared against the true coupling matrix.

Fieldtrip was used to calculate bivariate MI using their gcmi method (Oostenveld *et al* 2011).

Transfer Entropy: Transfer entropy (TE) utilizes Shannon entropy to quantify information transferred from one node to another (Schreiber 2000, Staniek and Lehnertz 2008) by calculating transition probabilities and determining whether including the history of an additional signal process improves the prediction of these probabilities. TE makes no linearity assumptions and provides directional information about interactions, though this comes at the expense of computational efficiency (Sabesan *et al* 2009, Vicente *et al* 2011). The absence of assumptions about the underlying relationships between nodes makes TE an attractive option for understanding biological systems that are not well characterized.

*Bivariate Transfer Entropy:* TE measures transition probabilities to evaluate directional information transfer. Consider the time series $\widehat{{x_m}}$, approximated by a stationary Markov process of order ${L_m}$, as defined above. The conditional probability of finding $\widehat{{x_m}}$ in state $\widehat{{x_m}}\left[ {k + 1} \right]$ at time sample $k + 1$ is calculated as $p\left( {\widehat{{x_m}}\left[ {k + 1} \right]|\widehat{{x_m}}\left[ k \right], \cdots ,\widehat{{x_m}}\left[ {k - {L_m} + 1} \right]} \right) = p\left( {\widehat{{x_m}}\left[ {k + 1} \right]|\widehat{{x_m}}\left[ k \right], \cdots ,\widehat{{x_m}}\left[ {k - {L_m}} \right]} \right)$.

We denote $\widehat{x_m^l}\left[ k \right] = \left[ {\widehat{{x_m}}\left[ k \right], \cdots ,\widehat{{x_m}}\left[ {k - {L_m} + 1} \right]} \right]$. The average number of bits needed to encode one additional state of, if all the previous states are known, is given by entropy rate:

\begin{align*}{h_{\widehat{{x_m}}}} &amp; = - \mathop \sum \limits_{k = 0}^{{k_f} - 1} p\left( {\widehat{{x_m}}\left[ {k + 1} \right],\widehat{x_m^l}\left[ k \right]} \right){\log _{10}} \nonumber\\ &amp; \quad \times p\left( {\widehat{{x_m}}\left[ {k + 1} \right]|\widehat{x_m^l}\left[ k \right]} \right).\end{align*}

For the two time series, it is preferable to measure the deviation from the generalized Markov property $p\left( {\widehat{{x_m}}\left[ {k + 1} \right]|\widehat{x_m^l}\left[ k \right]} \right) = p\left( {\widehat{{x_m}}\left[ {k + 1} \right]|\widehat{x_m^l}\left[ k \right],\widehat{x_n^l}\left[ k \right]} \right)$. Transfer entropy measures the influence of the states of $\widehat{{x_n}}$ on the transition probabilities of $\widehat{{x_m}}$, which we define:

\begin{align*}T{F_{n \to m}} &amp; = \mathop \sum \limits_{k = 0}^{{k_f} - 1} p\left( {\widehat{{x_m}}\left[ {k + 1} \right],\widehat{x_m^l}\left[ k \right],\widehat{x_n^l}\left[ k \right]} \right){\log _{10}} \nonumber\\ &amp; \quad \times \frac{{p\left( {\widehat{{x_m}}\left[ {k + 1} \right]|\widehat{x_m^l}\left[ k \right],\widehat{x_n^l}\left[ k \right]} \right)}}{{p\left( {\widehat{{x_m}}\left[ {k + 1} \right]|\widehat{x_m^l}\left[ k \right]} \right)}}.\end{align*}

TE measures the amount of uncertainty reduced in future values of ${\hat x_m}$ by knowing the past states of ${\hat x_n}$.

*Multivariate Transfer Entropy:* In a multivariate setting, time series information from the residual time series ${\tilde X_r}$ is also taken into account, and multivariate transfer entropy is calculated not only on the values of the source information ${\hat x_n}$ but also conditions on the information contained in the residual time series ${\tilde X_r}$. We define the time samples at $k$ of the residual time series ${\tilde X_r}$ by the set ${\hat x_r}\left[ k \right]$. To reduce computational time, the toolbox IDTxl reduces the number of nodes included in ${\hat x_r}$, only including relevant nodes, which we define here as ${\hat x_r}_{_f}$. Next, multivariate transfer entropy (mTE) is defined as (Schreiber 2000):

\begin{align*}T{F_{n \to m|\widetilde {{X_r}}}} &amp; = \mathop \sum \limits_{k = 0}^{{k_f} - 1} p\left( {{{\hat x}_m}\left[ {k + 1} \right],{{\hat x}^l}_m\left[ k \right],{{\hat x}^l}_n\left[ k \right],{{\hat x}_r}_{_f}\left[ k \right]} \right){\log _{10}} \nonumber\\ &amp; \quad \times \frac{{p\left( {{{\hat x}_m}\left[ {k + 1} \right]|{{\hat x}^l}_m\left[ k \right],{{\hat x}^l}_n\left[ k \right],{{\hat x}_r}_{_f}\left[ k \right]} \right)}}{{p\left( {{{\hat x}_m}\left[ {k + 1} \right]|{{\hat x}^l}_m\left[ k \right],{{\hat x}_r}_{_f}\left[ k \right]} \right)}}.\end{align*}

For this paper, the Information Dynamics Toolkit XL (IDTxl) toolbox was used to calculate both bivariate and multivariate transfer entropy. The JidtGaussianCMI estimator was used (Wollstadt *et al* 2019). We note that this method can take a very long time to calculate, which was a limiting factor for our analyses.

*Shuffled Networks:* To measure statistical significance of outcomes relative to the null hypothesis that the networks extracted are random, we created random network topologies and compared them to the true networks to generate a distribution for the null hypothesis. Shuffled arrays were randomly generated using the same protocol used to generate the original networks.

### 2.4. Runtime calculation

All runtime calculations measure the total runtime for the calculation when run on cores at the University of Minnesota’s Supercomputing Institute. These runtimes include the actual time to calculate the metrics but exclude formatting or saving data. Calculations were run on MSI’s Agate and Mangi clusters. Agate nodes have AMD 7763 processors, which run 2.5 double-precision teraflops per core at base frequency (Trader 2021). Mangi nodes have AMD ROME processors, which run 0.043 teraflops per core at base frequency (AMD EPYC^TM^ 7002 Series Processors, n.d.).

### 2.5. Network similarity measure

To evaluate the accuracy of network reconstructions, we utilized both cosine distance between the estimated and true coupling matrices and receiver operating (ROC) curves. Cosine distance was chosen because utilizing the continuous EC values allows us to evaluate similarity to our true networks without establishing a threshold for connection to binarize connectivity plots. Furthermore, EC metric outputs are arbitrarily scaled, meaning the measure of distance used must be scale invariant, and common euclidean distance measures cannot be used. It calculates the angle between two vectors, with smaller angles indicating greater similarity. Self-connections, represented by diagonal values in the coupling matrices, were excluded from the analysis in concordance with the traditional analyses for the field. The remaining values were flattened into a one-dimensional vector, and cosine distance was computed using MATLAB’s *pdist2* function. The cosine distance metric ranges from 0 to 2, where values closer to 0 indicate stronger agreement between the reconstructed and ground-truth networks.

ROC curves were selected because binarized representations are commonly used to interpret network metrics. ROC curves provide a threshold-independent evaluation of performance by characterizing the trade-off between sensitivity and specificity across all decision thresholds. This allows metrics to be compared objectively in terms of discriminability and robustness, rather than at a single, potentially arbitrary cutoff. Similar to the cosine distance analyses, self-connections were excluded from analysis. Matlab’s *perfcurve* function was used to calculate ROC areas under the curve (AUC). Possible AUC values range from 0 to 1, with values closer to 1 indicating better performance, and values greater than 0.5 indicating performance better than chance.

### 2.6. Statistics

Groups were compared with a Kruskal-Wallis test using Matlab’s kruskalwallis. If significant differences were identified, post-hoc Dunn-Sidak testing was done using Matlab’s multcompare. Bonferroni correction was performed to correct for multiple comparisons among groups. The number of comparisons was calculated by adding the number of metrics compared to the number of varied parameters in the model (i.e. 5 different node counts when parameter was varied + 10 EC metrics, so for statistics on node count data, = 0.05/15 = 0.003). As a result, the corrected alpha values for each condition are as follows: number of nodes = 0.003, number of time points = 0.00357, noise = 0.00277, and network coverage = 0.0025.

## 3. Results

**Figure 4.**
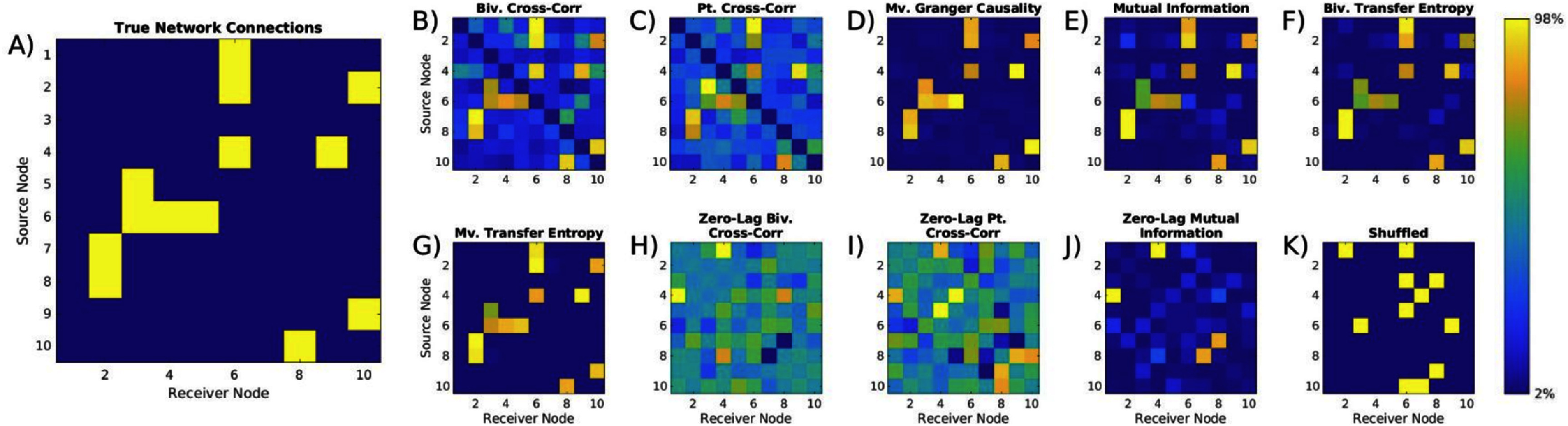
Example network reconstructions with effective connectivity metrics. Metrics are not on the same scale, so color scaling is set with the 2nd and 98th percentiles of values for each coupling matrix. (A) denotes the true network connections used to generate the time series data, where yellow identifies nodes are connected, while the following matrices are the effective connectivity between nodes calculated using (B) multi-lag bivariate (biv.) cross-correlation; (C) multi-lag partial cross-correlation; (D) multivariate (mv.) Granger causality; (E) multi-lag bivariate mutual information; (F) bivariate transfer entropy; (G) multivariate transfer entropy; (H) zero-lag bivariate cross-correlation; (I) zero-lag partial cross-correlation; (J) zero-lag bivariate mutual information; (K) Shuffled network with 10% coupling probability. In this figure, we observe an example network topology and metric network reconstructions of the exemplar network. We see that time-lagged metrics generate accurate reconstructions of the ten-node network, whereas zero-lag metrics visually fail to detect connections. This highlights the necessity of utilizing metrics that can successfully identify connections in systems where delays are present. Color represents effective connectivity between nodes as a percentile rank within each metric, scaled to the full range of effective connectivity values observed across this individual network.

We empirically evaluated the accuracy of several commonly used EC metrics to reconstruct simulated networks in the context of several practical limitations in intracranial electrophysiology data. An exemplar network topology of true network connectivity and its respective reconstructions can be seen in figure 4.

### 3.1. Number of nodes

Network size is crucial in intracranial electrophysiology, particularly as technological advances allow for greater numbers of intracranial electrodes to cover additional brain regions. To assess the impact of network size on reconstruction accuracy, we varied the number of nodes while holding the connection probability constant at 10%. The number of time points was fixed at 10 000 with no measurement noise.

**Figure 5.**
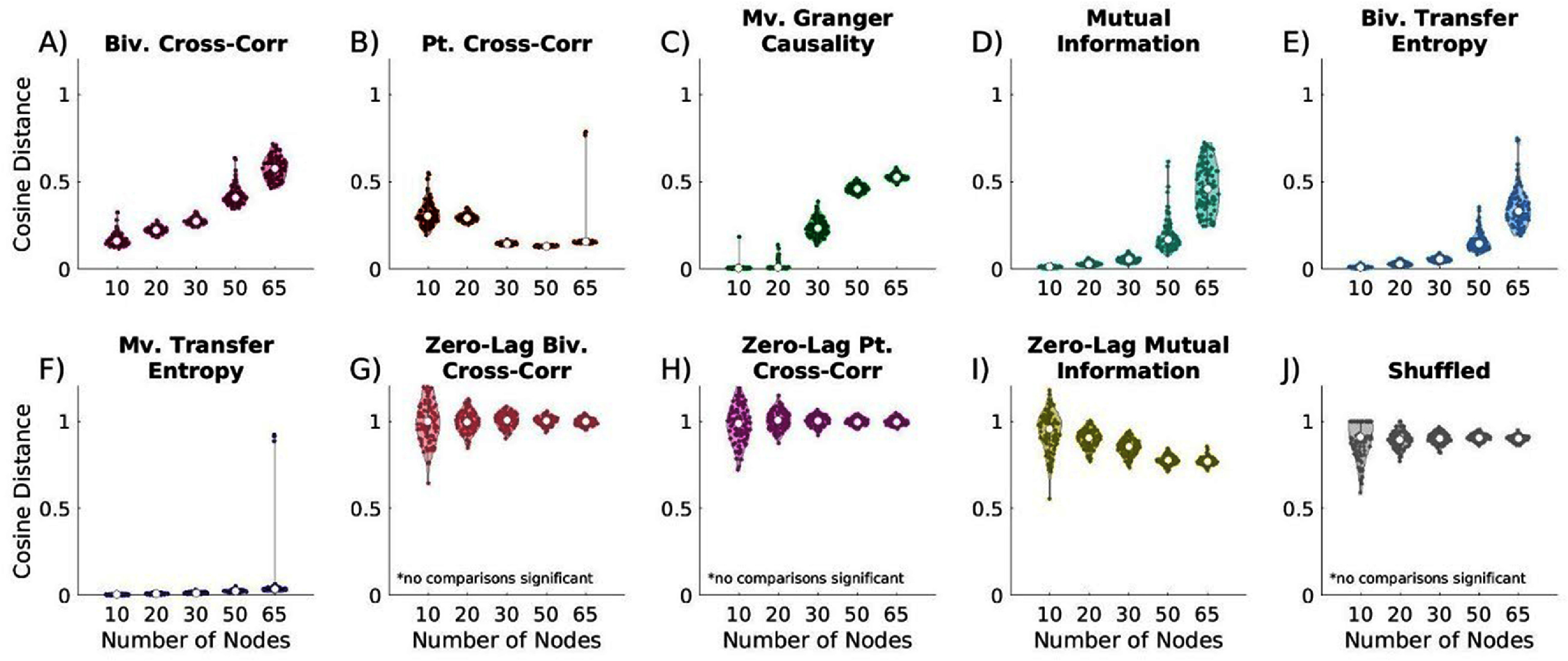
Effect of increasing network size on EC network reconstruction similarity to true networks. Cosine distance values close to 0 indicate the greatest possible similarity to the true networks. (A) time-lagged bivariate (biv.) cross-correlation, all comparisons are significant; (B) time-lagged partial (pt.) cross-correlation, all comparisons significant except 10 vs. 20 node networks; (C) time-lagged multivariate (mv.) Granger causality, all comparisons significant excluding 10 vs. 20 node networks; (D) time-lagged bivariate mutual information, all comparisons are significant; (E) time-lagged bivariate transfer entropy, all comparisons are significant; (F) time-lagged multivariate transfer entropy, all comparisons are significant; (G) zero-lag bivariate cross-correlation, no comparisons are significant; (H) zero-lag partial cross-correlation, no comparisons are significant; (I) zero-lag bivariate mutual information, all comparisons significant excluding 10 vs. 20 nodes and 50 vs. 65 nodes; (J) shuffled, no comparisons are significant. All lagged metrics worsen with increasing network size, excluding partial cross-correlation, which significantly improves as network size increases. For 10-20 node networks, multivariate Granger causality, bivariate transfer entropy, and multivariate transfer entropy perform comparably, while outperforming other metrics. For largest network sizes, multivariate transfer entropy and partial cross-correlation significantly outperform other metrics. Statistical comparisons are were completed with Kruskal-Wallis testing followed by Dunn-Sidak posthoc testing if significant differences were identified.

Increasing the network size worsened most time-lagged metrics, except partial cross-correlation, which improved with increasing network size (figure 4(B), p < 0.001). GC showed the most significant decline in similarity as network size increased (figure 5(C)). At the same time, multivariate transfer entropy (MVTE) exhibited only a slight but significant decline (figure 5(F)). For 10-node networks, multivariate GC, bivariate transfer entropy (BVTE), and MVTE performed similarly (Supplementary figure 1). However, as networks grew MVTE maintained strong performance, while GC and BVTE declined. As a result, multivariate transfer entropy outperformed GC and bivariate transfer entropy in larger networks (p < 0.001 for BVTE and GC in 50-node networks). Partial cross-correlation, conversely, performed poorly in small networks but improved and showed comparable performance to MVTE in networks with 50+ nodes, with both significantly outperforming other metrics.

Zero-lag metrics consistently underperformed or performed no better than shuffled networks across all network sizes when reconstructing networks with time-lagged relationships (figure 5(G)–(J)). These shuffled networks serve as a negative control, representing random networks with similar structure, establishing a baseline for comparison. In contrast, time-lagged metrics consistently outperformed shuffled networks. Additionally, multivariate versions of transfer entropy and cross-correlation generally outperformed their bivariate counterparts, with the performance gap becoming more pronounced in larger networks. This suggests that incorporating additional network-wide information enhances reconstruction accuracy particularly as network complexity increases. Results were mirrored in similar comparisons run using ROC curves to investigate sensitivity versus specificity relationship in evaluating networks (Supplementary figure 2)

### 3.2. Number of time points

**Figure 6.**
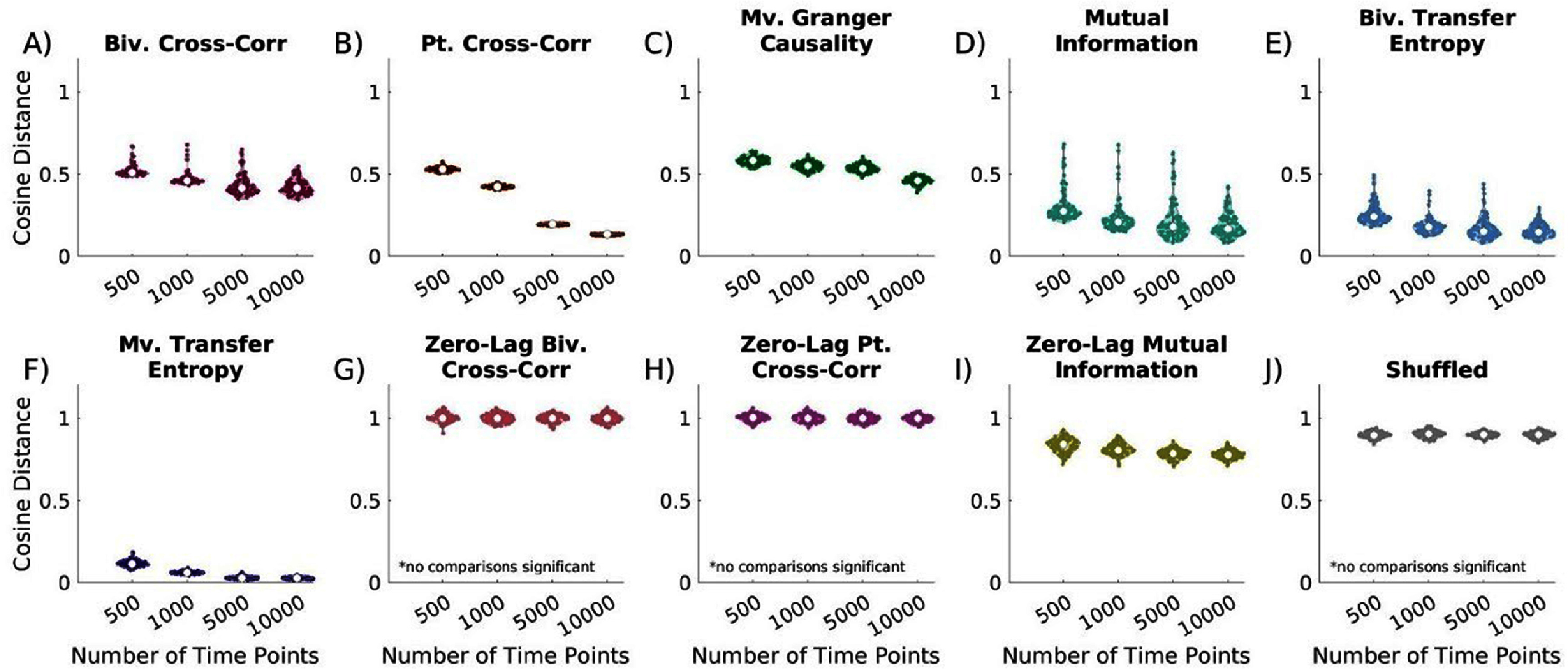
Effect of increasing time series length on EC network reconstruction similarity to true networks. Cosine distance values closest to 0 indicate the greatest similarity to the true networks. (A) time-lagged bivariate (biv.) cross-correlation, all comparisons significant except 5,000 vs. 10 000 time points; (B) time-lagged partial (pt.) cross-correlation, all comparisons significant; (C) time-lagged multivariate (mv.) GC, all comparisons significant except 1,000 vs 5,000 time points; (D) time-lagged bivariate mutual information, comparisons of 500 vs. all other numbers of time points are significant; (E) time-lagged bivariate transfer entropy, all comparisons significant except 1,000 vs 5,000 time points and 5,000 vs 10 000 time points; (F) time-lagged multivariate transfer entropy, all comparisons significant excluding 5,000 vs. 10 000 time points; (G) zero-lag bivariate cross-correlation, no comparisons significant; (H) zero-lag partial cross-correlation, no comparisons significant; (I) zero-lag bivariate mutual information, all comparisons significant except 5,000 vs. 10 000 time points; (J) shuffled, no comparisons significant. In general, network reconstruction significantly improves with increasing time series lengths. For all time series lengths multivariate transfer entropy is among the top performers. For 500 and 1,000 time points, bivariate and multivariate transfer entropy perform comparably. For 10 000 time points, partial cross-correlation performs comparably to multivariate transfer entropy. Statistical comparisons are were completed with Kruskal-Wallis testing followed by Dunn-Sidak posthoc testing if significant differences were identified.

Time series length is a critical factor in network reconstruction, as some studies rely on extended recordings while others analyze brief windows within behavior tasks. To assess its impact, we varied the number of time points in a fixed 50-node, measurement noise-free network. Increasing the number of time points improved all time-lagged metrics (p < 0.001 for 500 vs. 10 000 points, figures 6(A)–(F)), though the extent of improvement varied by metric. Most showed slight improvements, while partial cross-correlation improved significantly. Bivariate and multivariate transfer entropy performed similarly for shorter time series (500-1,000 points) (Supplementary figures 3(A) and (B), p = 0.067, p = 0.086). However, at 5,000 points, multivariate transfer entropy outperformed all other metrics (Supplementary figure 3(C)), and at 10 000 time points, multivariate transfer entropy and partial cross-correlation performed similarly (Supplementary figure 3(D), p = 0.012). Zero-lag metrics performed poorly in all conditions, and increasing time series length did not improve the accuracy of zero-lag bivariate or partial cross-correlation, underscoring that these metrics lack information about the true networks. Findings are similar in our ROC curve analysis, which also identifies reconstruction improvements with increasing numbers of time points (Supplementary figure 4).

### 3.3. Measurement noise

Noise is a common issue in neural recordings, often obscuring brain region and circuit activity while contaminating EC estimates (Bastos and Schoffelen 2016, Pesaran *et al* 2018). To evaluate its impact on network reconstructions, we introduced varying levels of measurement noise into time series data, altering the SNR. Simulations were conducted with 50 nodes, 10 000 time points, and a constant 10% connection probability.

All time-lagged methods, except bivariate cross-correlation, significantly improved as SNR increased (all p’s < 0.0001). In high-noise conditions, multivariate transfer entropy performed comparably to bivariate transfer entropy but outperformed all other metrics. When 5 ⩽ SNR ⩽ 10, multivariate transfer entropy consistently outperforms all other metrics. However, when SNR = 50, MVTE performs comparably to bivariate transfer and bivariate MI. Across all noise levels, bivariate transfer entropy performed similarly to partial cross-correlation and bivariate MI (Supplementary figure 5). Our ROC curve findings seen in Supplementary figure 6 paint a slightly different picture. While similarity to true networks may increase as SNR increases, nodes are still differentiable as connected versus not connected with ROC curves, indicating that connected nodes still demonstrate higher values than unconnected nodes when noise is non-correlated.

**Figure 7.**
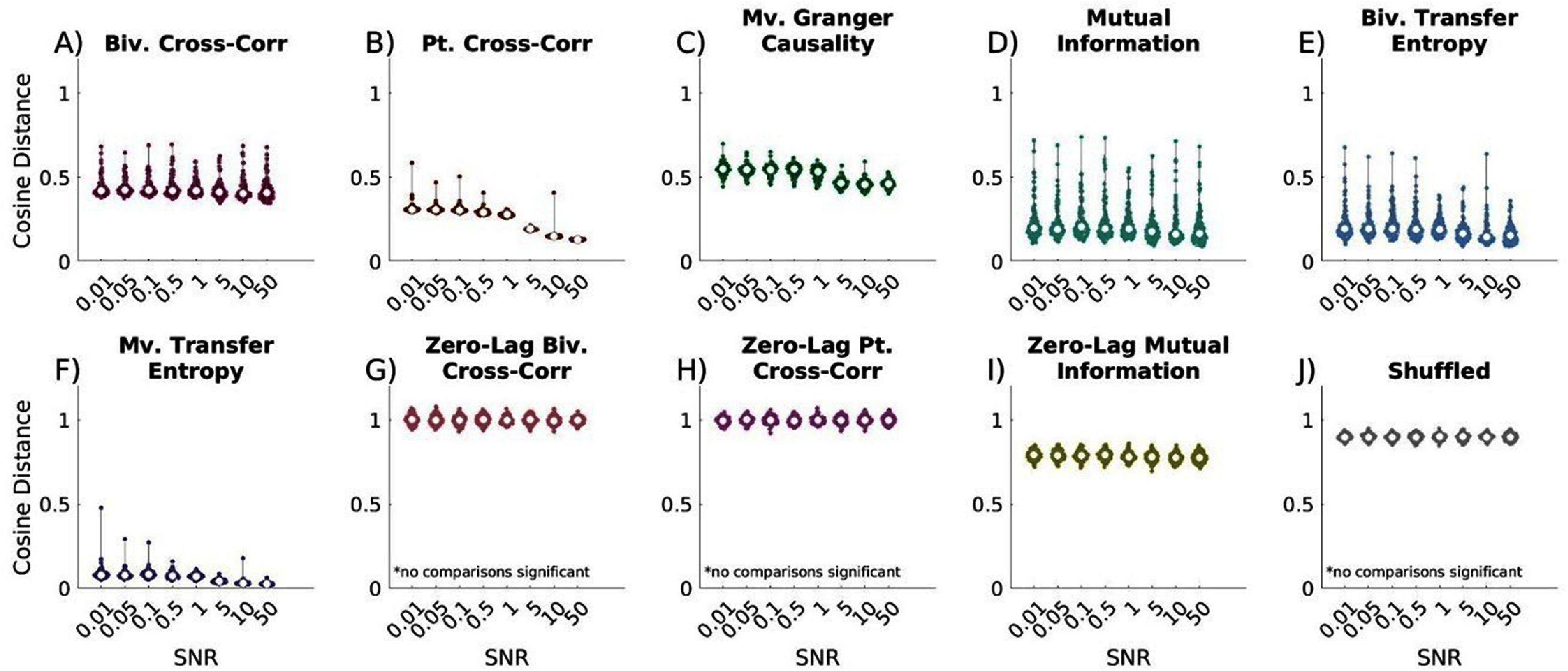
Effect of increasing Signal to Noise Ratio (SNR) on EC network reconstruction similarity to true networks. Cosine distance values closest to 0 indicate the greatest similarity to true networks. (A) time-lagged bivariate (biv.) cross-correlation, only SNR = 0.1 vs. 10 significant; (B) time-lagged partial (pt.) cross-correlation, all comparisons significant except SNR = 0.01 vs 0.05, 0.01 vs. 0.1, 0.05 vs. 0.1, 0.5 vs. 1, 5 vs. 10, and 10 vs. 50; (C) time-lagged multivariate (mv.) Granger causality, significant comparisons include SNR = 0.01 vs. 5, 0.01 vs. 10, 0.01 vs. 50, 0.05 vs. 5, 0.05 vs. 10, 0.05 vs. 50, 0.1 vs. 0.5, 0.1 vs. 5, 0.1 vs. 10, 0.1 vs. 50, 0.5 vs. 5, 0.5 vs. 10, 0.5 vs. 50, 1 vs. 5, 1 vs. 10, 1 vs. 50; (D) time-lagged bivariate mutual information, significant comparisons include SNR = 0.01 vs. 10, 0.01 vs. 50, 0.1 vs. 5, 0.1 vs. 10, and 0.1 vs. 50; (E) time-lagged bivariate transfer entropy, significant comparisons include SNR = 0.01 vs. 5, 0.01 vs. 10, 0.01 vs. 50, 0.05 vs. 5, 0.05 vs. 10, 0.05 vs. 50, 0.1 vs. 5, 0.1 vs. 10, 0.1 vs. 50, 0.5 vs. 10, 0.5 vs. 50, 1 vs. 5, 1 vs. 10, 1 vs. 50; (F) time-lagged multivariate transfer entropy, significant comparisons include SNR = 0.01 vs. 5, 0.01 vs. 10, 0.01 vs. 50, 0.05 vs. 5, 0.05 vs. 10, 0.05 vs. 50, 0.1 vs. 5, 0.1 vs. 10, 0.1 vs. 50, 0.5 vs. 5, 0.5 vs. 10, 0.5 vs. 50, 1 vs. 5, 1 vs. 10, 1 vs. 50, and 5 vs. 50; (G) zero-lag bivariate cross-correlation, no comparisons significant; (H) zero-lag partial cross-correlation, no comparisons significant; (I) zero-lag bivariate mutual information, significant comparisons include SNR = 0.01 vs. 10, 0.05 vs. 10, 0.1 vs.10, 0.5 vs. 10; (J) shuffled, no comparisons significant. All lagged metrics and zero-lag mutual information improve with increasing SNR. For low SNR conditions, multivariate transfer entropy significantly outperforms all other metrics, excluding bivariate transfer entropy, which performs comparably. As noise increases, multivariate transfer entropy begins to significantly outperform bivariate transfer entropy. This occurs when SNR = 0.1, 0.5, 5, 10, and 50. Otherwise multivariate transfer entropy significantly outperforms all other metrics in all conditions. Bivariate transfer entropy performs statistically no different than partial cross-correlation and mutual information for all noise levels. Statistical comparisons are were completed with Kruskal-Wallis testing followed by Dunn-Sidak posthoc testing if significant differences were identified.

As in previous tests, zero-lag metrics performed poorly at all noise levels, showing no significant difference from shuffled networks (figure 7(G)–(J)).

### 3.4. Network coverage

Coverage of brain networks in intracranial electrophysiology is often constrained by factors such as the limited intracranial space available for electrode placement, the cost of electrodes, the impracticality of high-density sampling, and hardware requirements for signal amplification. To assess how EC metrics performed under limited brain region coverage, we simulated partial network sampling using 50-node networks with 10 000 time points and no measurement noise. We progressively restricted the available data for reconstruction and compared the estimated connectivity to true connectivity.

**Figure 8.**
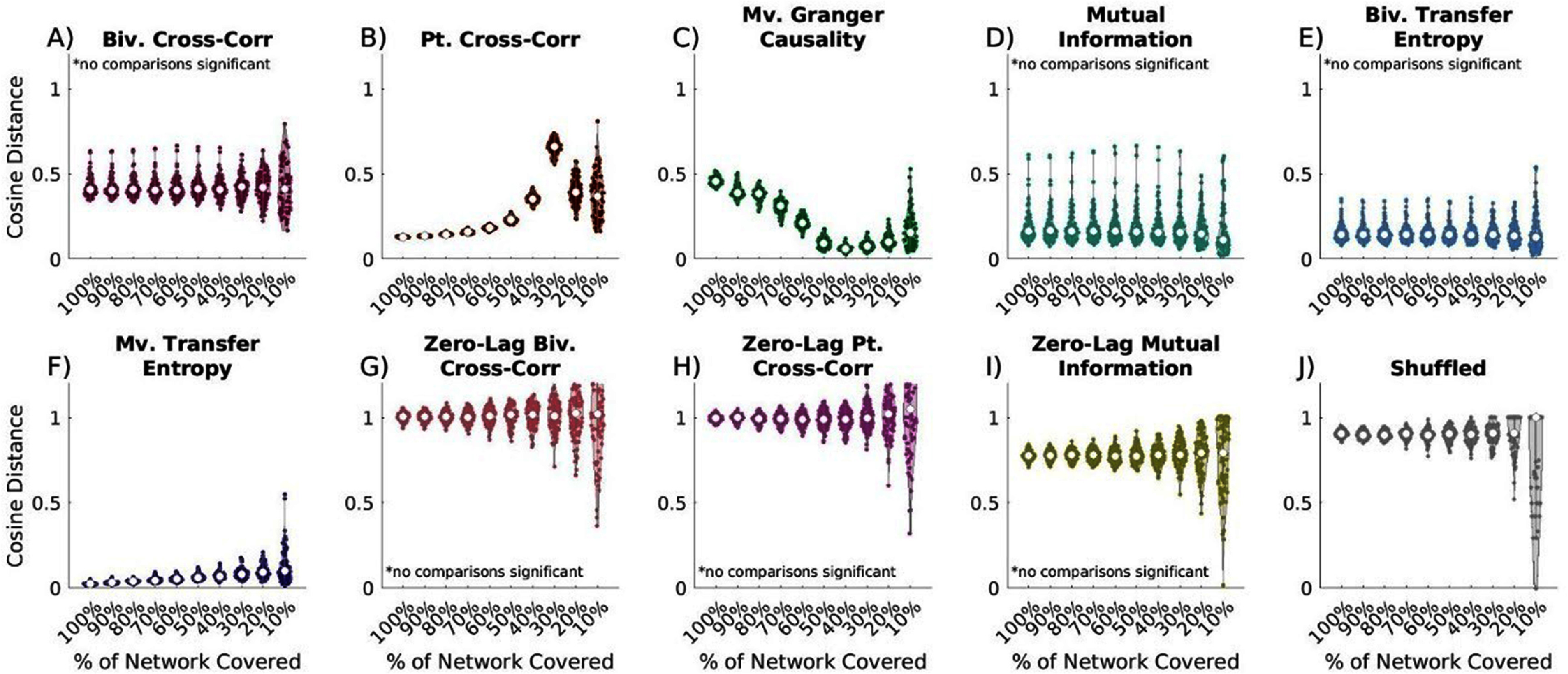
Effect of decreasing network coverage on EC network reconstruction similarity to true networks. Cosine distance values closest to 0 indicate the greatest possible similarity to the true networks. (A) time-lagged bivariate (biv.) cross-correlation, no comparisons significant; (B) time-lagged partial cross-correlation (pt.), significant comparisons include 100% vs 80%, 100% vs 70%, 100% vs 60%, 100% vs 50%, 100% vs 40%, 100% vs 30%, 100% vs 20%, 100% vs 10%, 90% vs 70%, 90% vs 60%, 90% vs 50%, 90% vs 40%, 90% vs 30%, 90% vs 20%, 90% vs 10%, 80% vs 60%, 80% vs 50%, 80% vs 40%, 80% vs 30%, 80% vs 20%, 80% vs 10%, 70% vs 50%, 70% vs 40%, 70% vs 30%, 70% vs 20%, 70% vs 10%, 60% vs 40%, 60% vs 30%, 60% vs 20%, 60% vs 10%, 50% vs 30%, 50% vs 20%, 40% vs 30%, and 30% vs 10%; (C) time-lagged multivariate (mv.) Granger causality, significant comparisons include 100% vs 70%, 100% vs 60%, 100% vs 50%, 100% vs 40%, 100% vs 30%, 100% vs 20%, 100% vs 10%, 90% vs 70%, 90% vs 60%, 90% vs 50%, 90% vs 40%, 90% vs 30%, 90% vs 20%, 90% vs 10%, 80% vs 60%, 80% vs 50%, 80% vs 40%, 80% vs 30%, 80% vs 20%, 80% vs 10%, 70% vs 50%, 70% vs 40%, 70% vs 30%, 70% vs 20%, 70% vs 10%, 60% vs 50%, 60% vs 40%, 60% vs 30%, 60% vs 20%, 40% vs 20%, 40% vs 10%, and 30% vs 10%; (D) time-lagged bivariate mutual information, no comparisons significant; (E) time-lagged bivariate transfer entropy, no comparisons significant; (F) time-lagged multivariate transfer entropy, all comparisons significant excluding 100% vs 90%, 90% vs 80%, 80% vs 70%, 70% vs 60%, 60% vs 50%, 50% vs 40%, 50% vs 30%, 50% vs 10%, and 40% network coverage compared to all lower percentages of coverage; (G) zero-lag bivariate cross-correlation, no comparisons significant; (H) zero-lag partial cross-correlation, no comparisons significant; (I) zero-lag bivariate mutual information, no comparisons significant; (J) shuffled, no comparisons are significant except 90% vs 10% coverage, 80% vs 10%, 70% vs 10%, 60% vs 10%, and 40% vs 10%. General patterns do not show large declines in the average reconstruction reliability for most metrics as the percentage of the network covered decreases. However, there are significant increases the variance of the reliability. Exceptions include partial cross-correlation which shows significant worsening in network reconstruction, and multivariate GC, which demonstrates significant improvement. Statistical comparisons are were completed with Kruskal-Wallis testing followed by Dunn–Sidak posthoc testing if significant differences were found.

Metric performance varied with declining network coverage. Bivariate cross-correlation, MI, and bivariate transfer entropy were unaffected (figure 8(A), (C) and (E)), although the variance of bivariate metrics increased significantly with declining network coverage (*p* < 0.0001). In contrast, partial cross-correlation (figure 8(B), *p* < 0.0001) and multivariate transfer entropy (figure 8(F), *p* < 0.0001) exhibited significant declines in performance. Interestingly, multivariate GC improved as network covered decreased (figure 8(D), *p* < 0.0001), suggesting that its sensitivity to spurious connections may be reduced in more sparsely sampled networks.

With 90%–100% coverage, multivariate transfer entropy and partial cross-correlation performed similarly (Supplementary figure 7, *p* = 0.0046, *p* = 0.0011), with MVTE significantly outperforming all other metrics. As coverage declined to 80–60%, MVTE remained the most accurate method, performing significantly better than all other metrics (*p* < 0.002). At 50% network coverage, GC matched the performance of MVTE, and at 10%, mutual information, GC, and bivariate transfer entropy performed comparably to multivariate transfer entropy (*p* < 0.0001). For most metrics (excluding mutual information), reducing network coverage increased variance in reconstruction accuracy (figure 8, *p*< 0.0001). Similar trends were observed in shuffled conditions, with small networks exhibiting high variance. Zero-lag metrics consistently underperformed, yielding worse results than time-lagged metrics and performing comparably to shuffled networks (*p* < 0.0001).

### 3.5. Runtime

The computational time required to calculate EC can impact a metric’s practical utility. Rapid computation is essential for the future of real-time applications, such as closed-loop neuromodulatory devices, whereas post-hoc analyses can tolerate slower methods. To assess efficiency, we measured the runtime for each metric, acknowledging that while absolute times may vary by computing environment, relative trends remain consistent.

**Figure 9.**
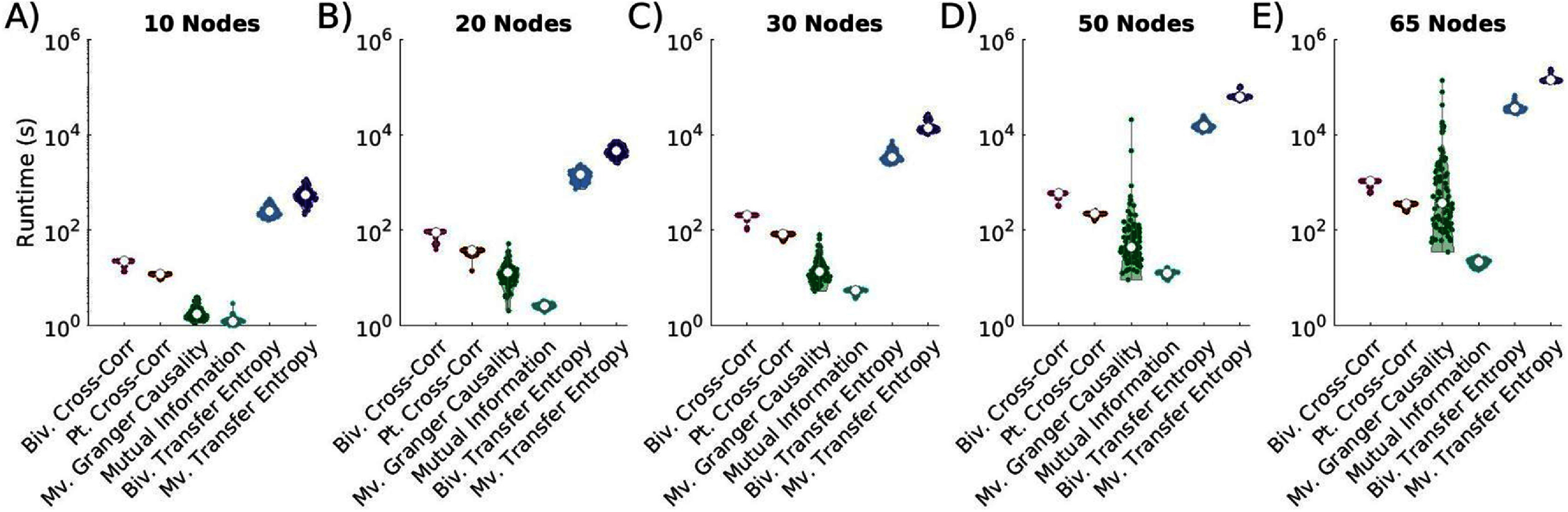
Comparative runtimes for EC metric calculation. Comparative runtimes on a log10-scale of time-lagged EC metrics, bivariate (biv.) cross-correlation (corr), partial (pt.) cross-correlation, multivariate (mv.) Granger causality, mutual information, bivariate transfer entropy, and multivariate transfer entropy for (A) 10, (B) 20, (C) 30, (D) 50, and (E) 65 node networks. Results show that mutual information consistently has the fastest runtime for calculation among metrics used for this analysis, while bivariate and multivariate transfer entropy consistently have the longest runtimes, which may be rate-limiting for online analyses and analyses of real data sets.

Despite its strong performance, MVTE is computationally intensive. For 10-node networks, MVTE averages 10 min to compute, compared to 20 s for bivariate cross-correlation (28 times faster) and 10 s for mutual information (475 times faster) (figure 9). As network size increases, TE-based runtimes scale inefficiently compared to other metrics. In 65-node networks, MVTE takes over 40 h to compute, while partial cross-correlation takes 15 min, and mutual information takes just 20 s—nearly 7000 times faster than MVTE. While bivariate transfer entropy also has a high computational cost, it is slightly faster than multivariate TE.

Among non-TE metrics, mutual information is the most efficient, while bivariate cross-correlation is the slowest. GC is often comparable to cross-correlation metrics.

## 4. Discussion

In these experiments, we examined several practical limitations encountered in intracranial electrophysiology recordings and their impact on the accuracy of network reconstructions. To accomplish this, we generated simulations of time series data with a VAR model using Erdős-Rényi connectivity. We then applied multiple EC metrics to infer network topology and evaluated their performance under different conditions.

In this study, our findings confirm that most EC metrics struggle to accurately reconstruct larger networks, with performance declining as the network size grows. The exception to this trend is partial cross-correlation, which improves with increasing network size. For small networks (<20 nodes), mutual information, GC, and MVTE perform well, though MVTE’s computational demands remain a significant limitation. However, as network size grows, the accuracy of mutual information and GC declines, while partial cross-correlation and MVTE maintain high performance, significantly outperforming other metrics in networks >20 nodes. GC’s poor performance with larger networks is noteworthy, as it relies on an autoregressive framework, yet fails to scale effectively. This is particularly notable as GC utilizes a VAR model to reconstruct networks, the same model used to generate time series for this analysis. This is the ideal condition under which GC should work and it is unlikely to perform better under more realistic conditions. Additionally, our results highlight that MVTE and partial metrics outperformed bivariate ones in networks of 30 nodes or more, emphasizing the importance of capturing multi-region interactions when analyzing large-scale brain networks.

### 4.1. Effect of network size

Interactions across brain networks are crucial for cognitive function, often requiring high-density electrode arrays such as electrocorticography or stereoelectroencephalography to capture widespread activity. As the size of the networks increase, it is essential that EC metrics remain reliable in reconstructing connectivity. Our findings confirm that most EC metrics struggle to accurately reconstruct larger networks, with performance declining as the network size grows. The exception to this trend is partial cross-correlation, which improves with increasing network size. We suspect that explicitly modeling the influence of additional nodes by partial cross correlation, such as indirect pathways, common drivers, and higher-order interactions, provides a richer representation of the complex structure of interactions between nodes, which in turn can improve metric performance. We see this in the contrast between the performance of partial cross correlation as compared to bivariate cross-correlation in larger networks. However, we do not see such an improvement in multivariate transfer entropy. In MVTE, baseline performance is already very high, even in relatively small networks. Consequently, limited improvement is possible for the reconstructions, producing a ceiling effect in performance. This contrasts with partial cross-correlation appears to be overly aggressive in small networks, where conditioning on a limited set of variables may remove variance from true interactions or inflate the apparent influence of a small number of relationships.

In conclusion, for small networks (<20 nodes), mutual information, GC, and MVTE perform well, though MVTE’s computational demands remain a significant limitation. However, as network size grows, the accuracy of mutual information and GC declines, while partial cross-correlation and MVTE maintain high performance, significantly outperforming other metrics in networks >20 nodes. GC’s poor performance with larger networks is noteworthy, as it relies on an autoregressive framework, yet fails to scale effectively. Additionally, our results highlight that MVTE and partial metrics outperformed bivariate ones in networks of 30 nodes or more, emphasizing the importance of capturing multi-region interactions when analyzing large-scale brain networks.

### 4.2. Time series length

To assess how the length of time series impacts network reconstruction, we varied the number of time points available for analysis. While some network reconstructions, such as those from resting state data, may analyze extended recordings, many studies may investigate network connectivity changes over the course of a window within a behavioral trial. In these cases, selecting an appropriate EC metric is crucial for ensuring reliable network inference. As expected, our results demonstrate that reconstruction accuracy improves for all EC metrics as the number of time points increases. Among these, partial cross-correlation shows the greatest improvement with increasing time series length, suggesting its sensitivity to longer datasets. MVTE maintains consistent performance across all recording lengths, performing comparably to bivariate transfer entropy for shorter time series (500-1000) points but excelling with longer recordings (10000 points). These results highlight MVTE’s reliability when reconstructing networks, even with short recordings. One limitation of this analysis is its focus on time series length, excluding the potential influence of sampling frequency on reconstruction accuracy, where a basic assumption is that the sampling rate is sufficient to capture the dynamics of the system.

### 4.3. Measurement noise

Brain activity recorded through electrophysiology is often contaminated by multiple sources of noise in the environment, including movement artifacts, particularly in the operating room or hospital, where the presence of other medical equipment may increase the amount and number of sources of noise. While researchers take many precautions to reduce this noise, understanding how these metrics perform under noisy conditions is critical. Previous studies have emphasized the importance of characterizing and removing noise before network analysis (Bastos and Schoffelen 2016, Pesaran *et al* 2018), and others have established that standard techniques for noise removal, such as global signal regression (Saad *et al* 2012) and filtering (Barnett and Seth 2011) may introduce biases or distort network reconstructions.

Our analysis models signals with independent measurement noise sources for each electrode and assumes correlated noise had been removed. Several key findings emerged. First, and unsurprisingly, reducing noise levels improves the performance of all metrics, reinforcing the importance of both hardware-based noise reduction and analytical denoising techniques to enhance reconstruction validity. Second, MVTE remains robust across all noise levels and outperforms all other metrics. This aligns with findings from other experimental conditions about the superiority of MVTE for accurate network reconstructions. However, our analysis is limited by the assumption that noise sources are uncorrelated across nodes or that correlated noises cannot be removed, which may not be true for neural data with volume conduction.

An interesting observation from these results is the difference in performance between partial cross-correlation and GC. Despite the fact that both are linear measures, their performance diverges as time series length and SNR increase. This is likely because they rely on fundamentally different estimation procedures and assumptions. With longer time series, partial cross-correlation benefits from improved estimation of covariance and partial covariance terms, and estimates converge rapidly. Partial cross-correlation also appears to be less sensitive to residual noise because it operates on second-order statistics and does not require explicit fitting of a dynamical model fitting beyond basic conditioning on other variables. Comparatively, GC is dependent on the appropriate estimation of autoregressive (AR or ARIMA) models. Even with high SNR and long recordings, performance is constrained by how well the chosen model order captures the true temporal dynamics. Misestimation of this model order or unmodeled temporal structures can degrade the metric’s performance. As a result, while both metrics improve with increased data length and increasing signal to noise ratio, partial cross-correlation likely outperforms GC because it leverages the additional samples without incurring the risks associated with parametric time-series model fitting. We suspect that the practical performance gap is driven less by linearity, and more by differences in estimator robustness and sensitivity to modeling assumptions.

### 4.4. Network coverage

In neural electrophysiology recordings, our electrodes often provide incomplete coverage of the networks of interest. Therefore, evaluating how EC metrics perform with missing data is essential for ensuring accurate network reconstructions. The impact of network coverage varies by metric. Bivariate cross-correlation, mutual information, and bivariate transfer entropy show minimal changes to average reconstruction reliability with declining coverage. MVTE exhibits slight declines but remains robust across all levels of coverage. In contrast, GC and partial cross-correlation are more affected. Interestingly GC improves as network coverage declines with performance peaking at 50% (25 nodes). This aligns with our network size results, which show that GC produces excellent network reconstructions for smaller networks of 10–20 nodes but poorly reconstructs larger networks of 30 nodes or larger. We see similar trends with declining network coverage in networks of other sizes, with GC reconstructions improving until the networks are 20 nodes or smaller (not shown), suggesting that declining network coverage is likely an artifact of GC’s inherent tendency to perform well for smaller networks given a fixed amount of time series data.

Conversely, partial cross-correlation worsens significantly with reduced coverage, and it performs poorest when only 30% (15 nodes) of the network is included in reconstruction. This is consistent with network size results, which demonstrate that partial cross-correlation is unreliable for networks with fewer than 20 nodes. The pattern remains consistent across networks of varying sizes (not shown), suggesting the issue stems from node count rather than percent coverage. We suspect that the appearance of improvement may be an artifact of the behavior of PCC when the conditioning set is severely underspecified. When only a small fraction of the network is included, the variance of the PCC estimate becomes extremely large. In this regime, the metric is dominated by a numerator-denominator imbalance. Because most of the network is omitted, both the residual covariance (the numerator) and the residual variance terms (the denominator) are driven toward their lower bounds. We suspect that this ‘bottoming out’ effect can sharpen contrasts between relationships, producing the illusion of improved performance despite the loss of true explanatory context. Therefore, this finding may be a marker that the large variance associated with such sparse coverage makes the estimates unstable and highly sensitive to noise. As a result, apparent performance gains observed at the very bottom of the coverage distribution should be interpreted skeptically, potentially as a statistical artifact rather than meaningful signal recovery.

Across all metrics, declining coverage also leads to greater variability in reconstruction accuracy, even for zero-lag metrics and shuffled data. This variability cannot be fully explained by network size changes, suggesting that missing nodes inherently increase reconstruction inconsistencies. One potential contributing factor not evaluated here may arise from the common input problem, where two nodes may share input from a single node. When not observed, multivariate methods may be unable to account for this connection, leading the EC metric to identify a spurious connection between two nodes that share an input. This is particularly relevant for intracranial networks, where studies show coverage is extremely limited, even in epilepsy patients with extensive montages (Katz and Abel 2019, Anderson *et al* 2021). These findings highlight the consistent reliability of EC metrics despite changes to the degree of network coverage, but the increased variability seen with declining coverage should lead us to question whether we can reliably interpret data where network coverage is so limited.

### 4.5. Zero-lag metrics

As expected, in networks with time-lagged relationships, zero-lag metrics (bivariate cross-correlation, partial cross-correlation, and bivariate mutual information) performed significantly worse than time-lagged metrics, but it is important to understand how poorly these metrics perform if time lags become nontrivial. In many cases, zero-lag metrics often fail to generate accurate networks, often performing no better than randomly shuffled network topologies when time series relationships are time-lagged. These findings highlight the necessity of using metrics that account for temporal delays in time-series data. In neural electrophysiology, where sampling rates often exceed the information transfer rates between brain regions, zero-lag metrics are unsuitable. Notably, there are modalities, such as fMRI, where sampling rates are low, and it is unclear that the above condition is met. In fMRI data, researchers must consider the expected communication lag between two regions, and whether communication-related changes may lag behind changes in the source by one sample or more. This issue becomes increasingly salient as fMRI methodologies continue to evolve and repetition times (TRs) decrease. Although zero-lag metrics may be appropriate for current widely used fMRI paradigms with TRs of approximately 1–2 s, this assumption will not hold as TRs continue to shorten. In sum, when network interactions unfold over timescales that exceed a single sampling interval, lagged metrics become critical for accurate network reconstruction.

These results also point to the importance of ensuring lag times for EC calculations are sufficiently long to encompass the communication lag between tested regions. If lags tested are shorter than the actual lag in communication between all region combinations, connections whose communication lag is shorter than the maximum tested window are likely to be identified, while regions with longer lags in communication are likely to be spuriously identified as not communicating. Exceptionally long lags are less likely to lead to problems with appropriate network reconstruction, however, addition of excess numbers of lags increases computational resource burden of calculating metrics. This time burden is variable per metric, but may be substantial depending on the metric used and the excess of additional lags included.

The above considerations underscore the importance of explicitly accounting for physiological communication delays between brain regions, as interactions may be systematically overlooked when analysis windows are too short to capture the temporal requirements of neural signal propagation.

### 4.6. Runtime

While MVTE outperforms other metrics in many contexts, its lengthy computational time limits its practical utility. For a 50-node network with 10 possible lags, TE averaged 17 h, compared to just 12.8 s for mutual information, the fastest metric. As TE runtimes increase significantly with more lags, analyzing 20–50 node networks with a 2000 Hz sampling rate and physiological lags could take weeks, making TE impractical for real-time applications. For real-time or closed-loop applications, mutual information seems to offer the best balance between speed and accuracy. Additionally, partial cross-correlation is a viable alternative for larger networks, achieving comparable reconstructions while averaging 220.2 s for the same 50-node networks. Although partial cross-correlation is not ideal for real-time use, implementing it in lower level programming languages could enhance its feasibility for online applications.

## 5. Limitations

The work in this project has a number of identifiable limitations. First, this study relies on simulated data rather than electrophysiological recordings, which limits the generalizability of our findings to neuronal systems. Furthermore, these time series are both Gaussian and stationary, and do not mimic features of neuronal electrophysiology, such as oscillatory activity or nonstationarities typical of neural recordings. Second, networks used to generate these simulations had Erdős–Rényi topologies, which lack the small-world or scale-free properties often observed in brain networks. Third, the relationships between time series in these analyses were modeled using first-order linear dynamics. While this assumption is prevalent in the literature, and much research exists proposing that large-scale neuronal relationships are likely to be linear (Blinowska and Malinowski 1991, Gultepe and He 2013, Nozari *et al* 2024), we cannot rule out the possibility of higher-order and nonlinear interactions. These simplifications were intentional, allowing us to construct the simplest possible model to systematically evaluate EC metrics under controlled conditions. Nonetheless, future work should expand to include real intracranial electrophysiology data, biologically inspired network topologies, and more complex temporal dependencies to better understand the robustness of these metrics under a broad variety of conditions.

## 6. Conclusion

EC metrics vary in their ability to reconstruct neural networks, making it crucial to understand their relative strengths and tradeoffs under different conditions. In this study, we systematically evaluated EC metrics on equal footing across key parameters, including network size, time series length, noise levels, and coverage, allowing for a direct comparison of their performance. Our results, summarized in table 2, show that multivariate transfer entropy provides the most accurate reconstructions overall, likely because this method is multivariate, nonlinear, and time-lagged, but its high computational cost limits its practical use in real-time applications. In contrast, mutual information and bivariate cross correlation offer efficient alternatives, excelling in small to medium-sized networks with rapid computation times that make it suitable for online analyses. Partial cross-correlation performs well in large networks when sufficient time series data is available, providing reliable reconstructions with significantly shorter runtimes than MVTE. Importantly, zero-lag metrics consistently fail when network interactions occur across multiple time lags, underscoring the necessity of using time-lag sensitive EC methods in neural electrophysiology. By evaluating these metrics under matched conditions, this work clarifies the tradeoffs between accuracy, computational efficiency, and suitability for different datasets and approaches.

**Table 2.** Summary of effective connectivity metric selection under variable data constraints. This table describes a set of decision points for selecting appropriate metrics based on the findings from this text.

	Capture time- lagged relationships	Perform well in small networks	Perform well in large networks	Perform well with low SNR	Perform well with high network coverage	Perform well with low network coverage	Reasonable calculation time
Bivariate cross correlation	+	+	—	—	—	—	+
Partial cross correlation	+	—	++	+	++	- -	+
Multivariate granger causality	+	++	—	—	—	+	+
Bivariate mutual information	+	++	—	++	+	+	++
Bivariate transfer entropy	+	++	—	++	+	++	−−
Multivariate transfer entropy	++	++	++	++	++	++	−−
Zero lag methods	−−	−−	−−	−−	−−	−−	+

## Data Availability

External Software: Functional Connectivity Toolbox: https://sites.google.com/site/functionalconnectivitytoolbox/. Multivariate Granger Causality Toolbox: www.mathworks.com/matlabcentral/fileexchange/78727-the-multivariate-granger-causality-mvgc-toolbox. Fieldtrip Mutual Information: https://github.com/fieldtrip/fieldtrip/blob/master/connectivity/ft_connectivity_mutualinformation.m. Information Dynamics Toolbox XL (IDTxl): https://github.com/pwollstadt/IDTxl. The data that support the findings of this study are openly available at the following URL/DOI: https://github.com/hermandarrowlab/ec-metrics-validation (Dembny *et al* 2026). Supplementary Data 1 available at https://doi.org/10.1088/1741-2552/ae5fd7/data1.
